# Multivariable Optimisation for Waiting-Time Minimisation at Roundabout Intersections in a Cyber-Physical Framework

**DOI:** 10.3390/s21123968

**Published:** 2021-06-09

**Authors:** Ovidiu Pauca, Anca Maxim, Constantin-Florin Caruntu

**Affiliations:** 1 Department of Automatic Control and Applied Informatics, “Gheorghe Asachi” Technical University of Iasi, 700050 Iasi, Romania; pauca.ovidiu@ac.tuiasi.ro (O.P.); anca.maxim@ac.tuiasi.ro (A.M.); 2 Holistic Engineering and Technologies (he[a]t), Continental Automotive Romania, 700671 Iasi, Romania

**Keywords:** roundabout intersection, cyber-physical system, waiting time minimisation, intelligent traffic systems

## Abstract

The evolution of communication networks offers new possibilities for development in the automotive industry. Smart vehicles will benefit from the possibility of connecting with the infrastructure and from an extensive exchange of data between them. Furthermore, new control strategies can be developed that benefit the advantages of these communication networks. In this endeavour, the main purposes considered by the automotive industry and researchers from academia are defined by: (i) ensuring people’s safety; (ii) reducing the overall costs, and (iii) improving the traffic by maximising the fluidity. In this paper, a cyber-physical framework (CPF) to control the access of vehicles in roundabout intersections composed of two levels is proposed. Both levels correspond to the cyber part of the CPF, while the physical part is composed of the vehicles crossing the roundabout. The first level, i.e., the edge-computing layer, is based on an analytical solution that uses multivariable optimisation to minimise the waiting times of the vehicles entering a roundabout intersection and to ensure a safe crossing. The second level, i.e., the cloud-computing layer, stores information about the waiting times and trajectories of all the vehicles that cross the roundabout and uses them for long-term analysis and prediction. The simulated results show the efficacy of the proposed method, which can be easily implemented on an embedded device for real-time operation.

## 1. Introduction

The main benefit of a roundabout is that it contributes to the reduction of the number of accidents, but the fluidity of the traffic can be negatively influenced, as it is an area that can cause the production of bottlenecks. Moreover, the emissions of CO${}_{2}$ increase with the number of vehicles. Therefore, various studies that analyse the efficiency of the roundabouts and other cross intersections, along with solutions to optimise them were proposed. In [1,2,3,4], the authors study the influence of the geometry of roundabouts on the number of accidents and show that the form of the roundabout directly influences this number. For example, the results from [5] prove that the angle between vehicles, which depends on the view of the drivers, and the velocity of the vehicles can negatively influence the number of crashes.

In [6], the authors proposed a solution based on a support vector machine to estimate the moment at which the vehicle leaves the roundabout using information related to the steering angle, steering velocity, and steering wheel position, and the solution has an efficiency of 95%. Furthermore, in [7], a study is proposed in which the traffic in a roundabout intersection is monitored, with the information about velocities of vehicles, steering wheel angle, geometry of the roundabout being obtained from vehicle routes. Moreover, the information includes the number of dynamic objects, presence of pedestrians, cyclists, motorcyclists and the distances between vehicles. Another solution based on intelligent methods is proposed in [8], where the vehicles are trained to cross a roundabout using the Q-learning algorithm. Furthermore, a solution based on fuzzy logic is proposed in [9] to compute the velocity for vehicles in order to ensure safe travel through the intersection and to decrease the emission of CO${}_{2}$. A solution that combines techniques of image processing and artificial intelligence to command the time when the vehicles can enter the roundabout is proposed in [10]. Another study [11], which is based on image processing, has the aim to ensure a safe entering into a roundabout for all the vehicles. In [12], an intelligent manager to determine the speed of the vehicles is proposed while considering a roundabout connected with a crossroad intersection. The obtained results illustrate an increase in the number of vehicles traversing the intersections. There were also studies performed that propose solutions to model and optimise the intersections using intelligent methods, such as Q-learning algorithms, fuzzy logic, neural networks, and neuro-inspired control [13,14,15,16,17,18].

Furthermore, there are solutions to model the traffic and to predict the vehicles flow through an intersection using Petri net models and neuro-inspired approaches [19,20]. Moreover, there are various studies in the literature that analysed the quantity of the CO${}_{2}$ emissions in the intersection [21,22,23,24]. These studies analyse different kinds of roundabouts (not signalised/signalised, with different forms and number of entries/exits) [25,26]. The results prove that repeated variations of velocity contribute to an increase in emissions. In [27], the performed study demonstrates that replacing the signalised intersections with roundabouts reduces the emission of CO${}_{2}$, but the NO${}_{x}$ value is always smaller in the signalised intersection. Furthermore, the variation of the velocity can influence the number of emissions. Another solution is proposed in [28] where, at each sample time, a matrix is built with the position of vehicles in the roundabout. The solution is supposed to find the velocity of vehicles to avoid the cases in which two or more vehicles are in the same position at the same time. Furthermore, there are analytical solutions to compute the time when the vehicles should enter the roundabout. In [29], a solution for a roundabout with only two entries/exits is proposed. In this case, a merging zone is considered in front of each entry. The entry time is determined so that if there are two vehicles from different entries that want to enter the merging area, then there can be only one vehicle in this zone. Otherwise, the vehicles can be both in the merging zone but have to maintain a safe distance between them. In [30], a manager for a roundabout intersection is proposed to determine the rate of acceleration/deceleration of vehicles to avoid the collision between them.

**Figure 1:**
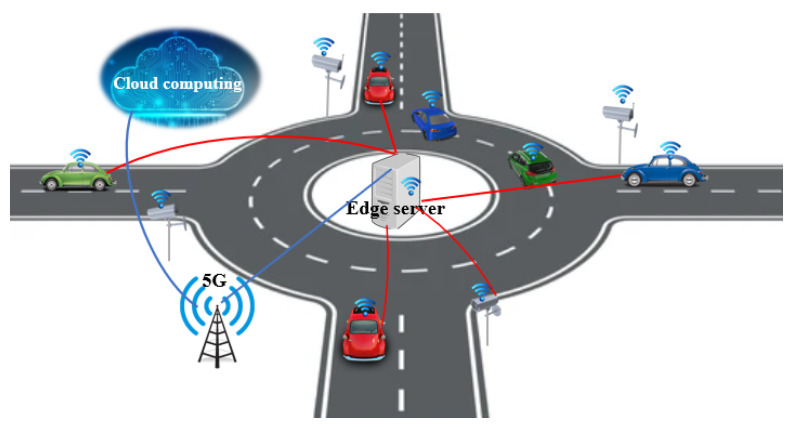
Cyber-physical roundabout manager.

Nowadays, the ideas of smart cities and autonomous/automated driving are strongly related to the interconnection of vehicles with the other traffic participants, smart infrastructure and cloud-computing platforms through the so-called cyber-physical frameworks (CPFs) [31]. This paper is a sequel to our previous work in [32], in which the problem of managing a roundabout with four entries/exits through minimising the waiting times of vehicles was firstly introduced. Moreover, safety constraints were also imposed and the problem was solved using the fmincon Matlab function. In the present work, the novelty is the generalisation of this problem, considering the intersection as having *n* entries/exits. The main contributions of the paper are the following: (i) proposing a cyber-physical framework to manage a roundabout intersection, the solution having two levels on the cyber plane, which are illustrated in Figure 1: the first level is represented by an edge server and the video cameras from the intersection, while the second level contains a cloud computing platform; (ii) the derivation of an analytical solution to minimise the waiting times of the vehicles and to ensure a safe drive through the roundabout. The first level, i.e., the edge-computing layer, interrogates the vehicles about their exits and receives information from the cameras installed on each entering lane—the data being used to compute the waiting times for vehicles. The second level, i.e., the cloud-computing layer, stores information received from the first layer about the waiting times and trajectories of all the vehicles that cross the roundabout and uses them for long-term analysis and prediction. The proposed solution is based on classical multivariable optimisation techniques, i.e., Lagrange multipliers method and Kuhn–Tucker conditions [33], to minimise the waiting times of vehicles. The same method is used in a manifold of applications to minimise a cost function with constraints, such as [34,35,36]. Among the applications that are based on this method, one can enumerate the following: trajectory planning for unmanned ground vehicles [37], optimal control of a diesel engine [38], minimisation of fuel consumption in a hybrid electric vehicle [39], control of dynamics of a robot manipulator [40] and thermal applications [41,42].

The main advantages of our proposed solution with respect to similar works are:

1. The consideration of a general case for a roundabout with *n* entries/exits; compared to the solutions in [29,30], we solve a general case of a roundabout intersection, and the solution does not depend on the number of entries/exits or number of vehicles;
2. The analytical formulation is easily implementable on an embedded device for real-time operation in a cyber-physical framework;
3. The methodology is suitable for fully automated and semi-automated vehicles;
4. The results show an increased number of vehicles crossing the roundabout in a certain period of time, when compared to our previous work [32];
5. The safety constraints are imposed for all the involved vehicles, i.e., the ones already inside the roundabout and vehicles that want to enter;
6. The CO${}_{2}$ emissions are reduced by eliminating the acceleration/deceleration of vehicles imposing a constant velocity;
7. Being a centralised solution, the vehicles do not have to be equipped with high computational power systems, which usually increase the costs too much.

**Notation and basic definitions**

Let R, $R_{+}^{*}$, Z and $Z_{+}$ denote the set of real, non-negative real, integer and non-negative integer numbers, respectively. $I_{n}\in R^{n\times n}$ denotes the identity matrix. Card(X) denotes the cardinal of the set *X* and is defined as the number of the elements of *X*.

## 2. Theoretical Foundation

In this section, classical multivariable optimisation techniques based on the Lagrange multipliers method and Kuhn–Tucker conditions are presented, setting the base of our solution using multivariable optimisation for waiting-time minimisation at roundabout intersections. The Lagrange multipliers method is mainly used to minimise a function with inequality and equality constraints, such as:

**Problem** **1.** *   Minimise f(X)*

*subject to*

(1) $$\left\{\begin{array}{c} q_{j}\left(X\right)\leq 0,j=1,2,\cdots ,m_{q},\\ g_{k}\left(X\right)=0,k=1,2,\cdots ,m_{g}, \end{array}\right.$$

*where $X={\left[x_{1},x_{2},\cdots ,x_{n}\right]}^{T}$ represents the vector of the optimisation variables, with n being their number, $q_{j}\left(X\right)\leq 0$ represents the inequality constraints, $g_{k}\left(X\right)=0$ represents the equality constraints, f, $g_{j}$, $g_{k}$, are derivable functions, $m_{q}$ and $m_{g}$ represent the number of inequality and equality constraints, respectively.*

The optimal solution for Problem 1 will be derived by applying the Kuhn–Tucker conditions:

(2) $$\left\{\begin{array}{c} \nabla f\left(X\right)+\sum_{j=1}^{m_{q}}\lambda_{qj}\nabla q_{j}\left(X\right)-\sum_{k=1}^{m_{g}}\lambda_{gk}\nabla g_{k}\left(X\right)=0,\\ \lambda_{qj}q_{j}\left(X\right)=0,\\ q_{j}\left(X\right)\leq 0,\;j=1,2,\cdots ,m_{q},\\ g_{k}\left(X\right)=0,\;k=1,2,\cdots ,m_{g},\\ \lambda_{qj}\geq 0,\\ \lambda_{gk}\geq 0, \end{array}\right.$$

where $\lambda_{qj}$ and $\lambda_{gk}$ denote the Lagrange multipliers associated with the constraints $q_{j}\left(X\right)\leq 0$ and $g_{k}\left(X\right)=0$, respectively. The gradient of function f(X) is denoted as ∇f(X). The Kuhn–Tucker conditions (2) represent the sufficient conditions of optimality, if *f* is a convex function and the constraints $q_{j}\left(X\right)$ and $g_{k}\left(X\right)$ are linear or convex functions [33].

At this point, the minimum solution and optimal points of specific functions will be determined, which will help to compute in Section 4 the minimum waiting times for the vehicles that want to enter the roundabout. These functions represent mathematical expressions for specific situations that can appear in a roundabout intersection, particularly:

- The roundabout is empty and only a vehicle wants to cross it;
- The roundabout is empty and *n* vehicles want to cross it;
- A vehicle wants to cross the roundabout and a vehicle is inside it.

In the remaining, the optimal solution of Problem 1 will be denoted with $X^{*}={\left[x_{1}^{*},x_{2}^{*},\cdots ,x_{n}^{*}\right]}^{T}.$

**Problem** **2.** *   Minimise $f\left(X\right)=\frac{1}{2}x_{1}^{2}$*

*subject to $x_{1}\geq 0$,*

*where $X=x_{1}$.*

*Solution:* It can be easily seen that min(f(X))=0 with $X^{*}=x_{1}^{*}=0$.

**Problem** **3.** *   Minimise $f\left(X\right)=\frac{1}{2}\sum_{i=1}^{n}x_{i}^{2}$*

*subject to $x_{i}\geq 0,i=1,\cdots ,n$,*

*where $X={\left[x_{1},\cdots ,x_{n}\right]}^{T}$.*

*Solution:* The gradient function f(X) can be computed as $\nabla f={\left[x_{1},\ldots ,x_{n}\right]}^{T}$, and from ∇f=0 yields that $x_{1}^{*}=x_{2}^{*}=\cdots =x_{n}^{*}=0$. The Hessian matrix is given by $H=I_{n}$, being positive definite (H>0), and from the fact that $x_{i}^{*}$ satisfies the constraint $x_{i}^{*}\geq 0,i=1,\ldots ,n$, yields that the optimal solution is obtained for $f(X^{*})=0$, where $X^{*}={\left[x_{1}^{*},x_{2}^{*},\cdots ,x_{n}^{*}\right]}^{T}$.

**Problem** **4.** *   Minimise $f\left(X\right)=\frac{1}{2}x_{1}^{2}$*

*subject to*

(3) $$\left\{\begin{array}{c} |c-x_{1}|\geq x_{s}\\ x_{1}\geq 0. \end{array}\right.$$

where $X=x_{1}$, $x_{s}\in R_{+}^{*}$, and c∈R.

The variable *s* can be defined as:

(4) $$s=sgn\left(x\right)=\left\{\begin{array}{c} +1,x>0\\ \;0,x=0\\ -1,x<0 \end{array}.\right.$$

*Solution:* The first constraint can be rewritten as: $|c-x_{1}|\geq x_{s}\Leftrightarrow$$sgn\left(c-x_{1}\right)\left(c-x_{1}\right)\geq x_{s}\Leftrightarrow$ $s_{1}\left(c-x_{1}\right)\geq x_{s}\Leftrightarrow$$x_{s}+s_{1}x_{1}-s_{1}c\leq 0$, $s_{1}=sgn\left(c-x_{1}\right)$. We used that |a|=sgn(a)∗a, a∈R. The second constraint can be rewritten as $-x_{1}\leq 0$. Now, the constraints $|c-x_{1}|\geq x_{s}\Leftrightarrow$$x_{s}+s_{1}x_{1}-s_{1}c\leq 0$ and $x_{1}\geq 0\Leftrightarrow$$-x_{1}\leq 0$ are rewritten according to the constraints of Problem 1. Thus, Problem 4 can be rewritten as:

**Problem** **5.** *   Minimise $f\left(X\right)=\frac{1}{2}x_{1}^{2}$*

*subject to*

(5) $$\left\{\begin{array}{c} q_{1}=x_{s}+s_{1}x_{1}-s_{1}c\leq 0\\ q_{2}=-x_{1}\leq 0. \end{array}\right.$$

where $X=x_{1}$, c∈R, $x_{s}\in R_{+}^{*}$ and $s_{1}\in \left\{-1,0,1\right\}$ are considered constants.

Knowing that *f* is a convex function $\left(f^{{}^{\prime \prime }}\left(x_{1}\right)=1>0,\forall x_{1}\in R\right)$ and the constraints $q_{1}\left(x_{1}\right)$ and $q_{2}\left(x_{1}\right)$ are linear functions $\forall x_{1}\in R$, the solution that satisfies the Kuhn–Tucker conditions is a solution for Problem 5. To solve Problem 5, the Kuhn–Tucker conditions given in (2) are applied:

(6) $$\left\{\begin{array}{c} \nabla f+\lambda_{1}\nabla q_{1}+\lambda_{2}\nabla q_{2}=0,\\ \lambda_{1}q_{1}=0,\\ \lambda_{2}q_{2}=0,\\ q_{1}\leq 0,\\ q_{2}\leq 0,\\ \lambda_{1}\geq 0,\\ \lambda_{2}\geq 0, \end{array}\right.$$

where $\lambda_{1}$ and $\lambda_{2}$ represent the Lagrange multipliers. Computing $\nabla f=x_{1}$, $\nabla q_{1}=s_{1}$, $\nabla q_{2}=-1$, (6) becomes:

(7) $$\left\{\begin{array}{c} x_{1}+\lambda_{1}s_{1}-\lambda_{2}=0,\\ \lambda_{1}\left(x_{s}-s_{1}c+s_{1}x_{1}\right)=0,\\ \lambda_{2}\left(-x_{1}\right)=0,\\ x_{s}-s_{1}c+s_{1}x_{1}\leq 0,\\ -x_{1}\leq 0,\\ \lambda_{1}\geq 0,\\ \lambda_{2}\geq 0. \end{array}\right.$$

The solution can be computed for several cases depending on $\lambda_{1}$ and $\lambda_{2}$. The first case will be represented by $\lambda_{1}=0$, the second case by $\lambda_{2}=0$ and the third one by $\lambda_{1}\neq 0$ and $\lambda_{2}\neq 0$. Next, each of the three cases will be analysed in detail.

(8) $$\;\mathrm{Case}1:\lambda_{1}=0\Rightarrow \left\{\begin{array}{c} x_{1}+\lambda_{2}=0\Rightarrow x_{1}=\lambda_{2},\\ -\lambda_{2}x_{1}=0,\\ x_{s}-s_{1}c+s_{1}x_{1}\leq 0,\\ -x_{1}\leq 0,\\ \lambda_{2}\geq 0. \end{array}\right.$$

The first and second equations in (8) yield that $x_{1}=\lambda_{2}=0$ and from the third one we have: $x_{s}-s_{x}c\leq 0\Leftrightarrow$ $x_{s}\leq s_{1}c$. From the constraint $|c-x_{1}|\geq x_{s}$, (3), and $x_{s}>0$ results that the solution $x_{1}=c$ is not a solution for Problem 4 ($x_{1}=c\Rightarrow 0\geq x_{s}$ but, $x_{s}>0\Rightarrow$ contradiction). Therefore, $s_{1}=sgn\left(c-x_{1}\right)\in \left\{+1,-1\right\}$. Thus, $x_{1}=\lambda_{2}=0$ if:

(9) $$x_{s}\leq c\;\mathrm{OR}\;x_{s}\leq -c$$

Considering $\lambda_{2}=0$ in (7), the second case results:

(10) $$\;\mathrm{Case}2:\lambda_{2}=0\Rightarrow \left\{\begin{array}{c} x_{1}+\lambda_{1}s_{1}=0,\\ \lambda_{1}\left(x_{s}-s_{1}c+s_{1}x_{1}\right)=0,\\ x_{s}-s_{1}c+s_{1}x_{1}\leq 0,\\ -x_{1}\leq 0,\\ \lambda_{1}\geq 0. \end{array}\right.$$

(11) $$\begin{array}{c} \;\mathrm{Subcase}2.1:\lambda_{1}=0\Rightarrow x_{1}=0\Rightarrow \mathrm{Case}\;1 \end{array}$$

(12) $$\;\mathrm{Subcase}2.2:\;\lambda_{1}\neq 0$$

(13) $$\begin{array}{c} \mathrm{Subcase}2.2.1:\mathrm{supose}\;x_{1}=0\Rightarrow \\ \Rightarrow \lambda_{1}=0\;\left(\mathrm{contradiction}\right) \end{array}$$

(14) $$\begin{array}{c} \;\mathrm{Subcase}2.2.2:\mathrm{supose}\;x_{1}\neq 0\Rightarrow \\ \Rightarrow \left\{\begin{array}{c} x_{1}=-\lambda_{1}s_{1},\\ x_{s}-s_{1}c+s_{1}x_{1}=0,\\ x_{s}-s_{1}c+s_{1}x_{1}\leq 0,\\ \lambda_{1}\geq 0,\\ -x_{1}\leq 0. \end{array}\right. \end{array}$$

The second equation in (14) yields that $x_{1}=c-x_{s}/s_{1}$ and replacing $x_{1}$ in the first and third equations from (14) we obtain that: $\lambda_{1}=-c/s_{1}+x_{s}/s_{1}$ and the third equation is accomplished as an equality from which yields $\lambda_{2}=0$. We used $s_{1}s_{1}=s_{1}/s_{1}=1$. If $s_{1}=1$ then $x_{1}=c-x_{s}$, $\lambda_{1}=-c+x_{s}$. Imposing the conditions $\lambda_{1}>0$ and $-x_{1}<0$ results: $c<x_{s}$ AND $c>x_{s}$, which yields no solution for $x_{1}$. If $s_{1}=-1$ then $x_{1}=x_{s}+c$, $\lambda_{1}=x_{s}+c$. Imposing the conditions $\lambda_{1}>0$ and $-x_{1}<0$ results that $x_{s}>-c$.

(15) $$\begin{array}{c} \;\mathrm{Case}3:\lambda_{2}\neq 0,\lambda_{1}\neq 0,\;\mathrm{then}\;\mathrm{from}\;\left(7\right)\Rightarrow \\ \Rightarrow x_{1}=0\;\mathrm{with}\;x_{s}=\pm c. \end{array}$$

In conclusion, there are two main solutions for Problem 4:

(16) $$\;\;\mathrm{Solution}\;1\text{:}\;\left\{\begin{array}{c} x_{1}^{*}=0\\ \lambda_{1}^{*}=0\;\mathrm{if}\;\left(x_{s}\leq c\right)\;\mathrm{OR}\;\left(x_{s}\leq -c\right)\\ \lambda_{2}^{*}=0 \end{array}\right.$$

(17) $$\mathrm{Solution}\;2\text{:}\;\left\{\begin{array}{c} x_{1}^{*}=x_{s}+c\\ \lambda_{1}^{*}=x_{s}+c\;\mathrm{if}\;\left(x_{s}>-c\right)\\ \lambda_{2}^{*}=0 \end{array}\right.$$

It can be observed that the results (16) and (17) represent all the possible solutions of Problem 4.

## 3. Problem Definition

This section presents the following interest points:

- The roundabout intersection description and the rules that manage it, extending the features introduced in our previous work [32];
- The cost function and safety constraints imposed to ensure both a safe entering into the roundabout and a minimum waiting time.

### 3.1. Roundabout Cyber-Physical Framework—General Information

**Figure 2:**
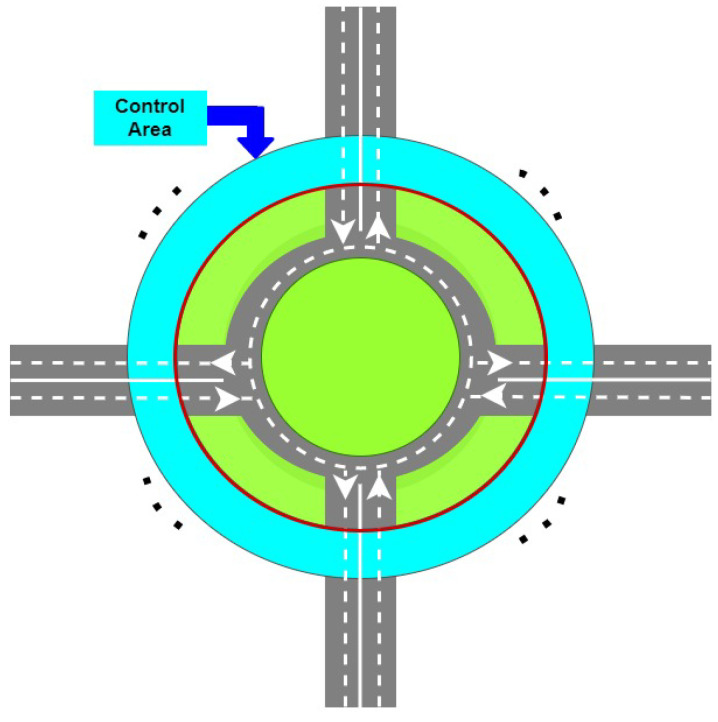
Roundabout intersection (*n* entries/exits).

A roundabout with *n* entries and exits, each of them having a single lane for both directions, is illustrated in Figure 2. The direction of the vehicles that are entering and leaving the intersection is counter-clockwise. The roundabout is controlled through a cyber-physical framework composed of two levels on the cyber plane, while the vehicles that cross the roundabout form the physical plane. As previously mentioned, the first level uses an edge-computing server with the task of a centralised manager (CM) to communicate with the vehicles to obtain data from the installed cameras and send information about traffic to the cloud platform. Using this data, the CM will compute the waiting times for the vehicles that want to enter the roundabout to avoid all collisions. The second level, which is based on a cloud-computing platform, receives traffic data from the CM, stores it and performs long-term analysis and prediction regarding the management of the roundabout.

The control area (CA) is the zone represented with turquoise. In this area, the CM asks each vehicle about its requested exit and sends to it the corresponding waiting time. If the vehicles are not yet interrogated, then they will stop and wait in the waiting-point represented by the intersection of the red circle with each entering lane until they receive the waiting time. Furthermore, after the waiting time is received, the vehicles have to stay in the waiting-point a period of time equal to their waiting time.

The CM computes the waiting time for the first vehicle from each entry in the control area, then it waits until they all enter the roundabout, marked with the grey circle. After that, it computes the waiting time for the next vehicles. This rule ensures equal priority for each entry, and the cases in which some entries are disadvantaged, thus having a higher waiting time, are excluded. By defining the cost function as a sum of all waiting times for the vehicles from each entry, a minimum global waiting time is ensured.

The main reasons for using a CM are:

- The vehicles do not have to be equipped with complex algorithms to ensure a safe entry in the roundabout;
- A high computational power unit for the vehicles is not required, which reduces the production costs;
- The CM is a system used for a whole intersection that can benefit from a high computational power unit without increasing the costs;
- The CM also employs all the information in the immediate vicinity of the roundabout, thus ensuring a global optimal solution and safety for all traffic participants.

In this work, all vehicles are considered fully automated and are moving with constant velocity, imposed by the roundabout rules. This assumption ensures economic traffic, knowing that a roundabout is a place with conglomerate traffic and the acceleration/deceleration produces more pollution due to high fuel consumption [25].

The proposed solution can be extended to vehicles that are semi-automated or driver-driven. The vehicles can communicate with the CM using different devices like navigation systems or smartphones together with a dedicated application. These can be used for sending to the CM the desired exit. After that, the CM will send to the device the waiting time and the driver can see it. When the driver arrives at the waiting-point, the application from the device starts to count a time period equal to the received waiting time. The application will announce to the driver when the waiting time has finished such that they can safely enter the roundabout. Following, the driver has to enter and cross the roundabout with a velocity as close as possible to the imposed velocity. If the vehicles are equipped with cruise control (CC) or adaptive cruise control (ACC) functionalities, this last issue is solved, and these functionalities ensure that the vehicle moves with the desired imposed velocity.

Our solution relies on existing vehicle-to-infrastructure (V2I) communication architecture with the standard IEEE802.11p for Dedicated Short-Range Communication (DSRC) [43]. The information can also be sent via wireless communication using the 4G/5G cellular interface or satellite links [44].

### 3.2. Multivariable Optimisation Problem with Safety Constraints

Hereafter, the cost function and the safety constraints are described, followed by the definition of the problem to be solved.

The time a vehicle spends in the roundabout depends on the waiting time and the distance between the vehicle entry and exit:

(18) $$\tau_{i}^{f}-\tau_{i}^{0}=\tau_{i}^{w}+\tau_{i}^{in}+\delta_{i}/v,$$

where $\tau_{i}^{f}$ represents the time when the vehicle $i\in Veh_{IN}$ leaves the intersection, $\tau_{i}^{0}$ represents the moment when the CM has the first contact with the vehicle *i*, $\tau_{i}^{w}$ is the waiting time to be computed by the CM, $\tau_{i}^{in}$ is the time needed by the vehicle to travel the distance between the point when it has the first contact with the CM and the point when it enters on the lane of the roundabout, and the last term, $\delta_{i}/v$, represents the time needed by the vehicle to travel the distance $\delta_{i}$, between its entry and exit at the imposed velocity *v*. Note that, $Veh_{IN}=\left\{1,2,\cdots ,n_{k}\right\}$ is the array of vehicles for which the CM has to compute the waiting time at step time *k*, $n_{k}\leq n$, where *n* represents the number of entries/exits in the roundabout.

However, the proposed solution can be applied for a general case of a roundabout because it is not developed for a specific number of entries/exits of the roundabout, diameter or lane width. The dimensions of the roundabout are only required when the CM has to compute the distance to be covered by a vehicle between an entry and the desired exit.

To reduce the time each vehicle spends in the roundabout, the value of the difference represented by Equation (18) has to be minimised. In this equation, the terms $\tau_{i}^{in}$ and $\delta_{i}/v$ cannot be modified and are considered as known constants, resulting in that only the term $\tau_{i}^{w}$ has to be minimised. Based on this, the cost function to be minimised by the CM, can be formulated as:

(19) $$J\left(\left[\tau_{1}^{w},\cdots ,\tau_{n_{k}}^{w}\right]\right)=\frac{1}{2}\sum_{i\in Veh_{IN}}{\left(\tau_{i}^{w}\right)}^{2}.$$

At this point, the safety constraints have to be imposed to ensure that the vehicles will enter the roundabout without collisions:

- The first constraint is imposed for the vehicles that want to enter the roundabout with respect to the vehicles that have already entered:
  (20) $$|\tau_{zi}-\tau_{i}^{in}-\tau_{i}^{w}|\geq \tau_{safety}$$
  where $\tau_{zi}$ is the time needed by the vehicle $z\in Veh_{IN_{zi}}$ to arrive in front of $E_{i}$, i.e., the entry for vehicle $i\in Veh_{IN}$, $Veh_{IN_{zi}}$ is the set of the vehicles that are already inside the roundabout and will pass in front of entry $E_{i}$, and $\tau_{safety}$ represents the safety time and corresponds to the imposed safety distance between vehicles $\delta_{safety}$, computed as $\tau_{safety}=\delta_{safety}/v$.
  This constraint ensures that the vehicles $i\in Veh_{IN}$ and $z\in Veh_{IN_{zi}}$ will not be in front of $E_{i}$ at the same time.
- The second constraint takes into account the vehicles $j,i\in Veh_{IN}$ with i≠j:
  (21) $$|\tau_{j}^{in}+\tau_{j}^{w}+\tau_{ji}-\tau_{i}^{in}-\tau_{i}^{w}|\geq \tau_{safety},$$
  where $\tau_{ji}$ is the time needed by vehicle $j\in Veh_{IN}$, i≠j, to arrive in front of entry $E_{i}$ of vehicle $i\in Veh_{IN}$.
  In this case, the constraint ensures that the vehicles *i* and *j* will not be at the same time in front of $E_{i}$, if vehicle *j* has to pass in front of this entry.
- The last constraint ensures that the waiting time is positive:
  (22) $$\tau_{i}^{w}\geq 0.$$

The CM will compute the waiting time in order to minimise the cost function (19) while satisfying all the constraints (20)–(22), defined as the following problem:

**Problem** **6.** *   Minimise (19)*

*subject to: (20)–(22).*

## 4. Main Theoretical Contribution

This section presents the analytical solution for Problem 6. The waiting times are determined using the theoretical solutions provided in Section 2.

**Case 1**: The first case is the one in which there are no vehicles inside the intersection, $Veh_{IN_{zi}}=⌀$, and only $n_{k}\leq n$ vehicles want to enter the roundabout. In this case, Problem 6 will be solved using the solution of Problem 3. Considering $\mathrm{Card}\left(Veh_{IN}\right)=n_{k}$, $\mathrm{Card}(Veh_{IN_{ji}})\neq 0$, $\mathrm{Card}(Veh_{IN_{zi}})=0$, Problem 6 becomes:

(23) $$\left\{\begin{array}{c} \min_{\left[\tau_{1}^{w},\cdots ,\tau_{n_{k}}^{w}\right]}\left(J\left(\left[\tau_{1}^{w},\cdots ,\tau_{n_{k}}^{w}\right]\right)\right)=\min_{\left[\tau_{1}^{w},\cdots ,\tau_{n_{k}}^{w}\right]}\sum_{i=1}^{n_{k}}\frac{1}{2}{\left(\tau_{i}^{w}\right)}^{2}\\ \;\mathrm{subject}\;\mathrm{to}\text{:}\;\tau_{i}^{w}\geq 0,i=1,\cdots ,n_{k} \end{array}\right.$$

In this case, the solution is $\tau_{i}^{w*}=0$, with $J^{*}=0$, $i=1,\cdots ,n_{k}$. The set $Veh_{IN_{ji}}$ includes the vehicles $j\in Veh_{IN}$ that will pass in front of entry $E_{i}$, i≠j.

**Case 2**: The next situation is represented by the case in which there is a vehicle that wants to enter the intersection through entry $E_{i}$ and in the roundabout there is only a vehicle that will pass in front of this entry. Considering $\mathrm{Card}(Veh_{IN})=1$, $\mathrm{Card}(Veh_{IN_{ji}})=0$, $\mathrm{Card}(Veh_{IN_{zi}})=1$, Problem 6 becomes:

(24) $$\left\{\begin{array}{c} \min_{\tau_{i}^{w}}\left(J\left(\tau_{i}^{w}\right)\right)=\min_{\tau_{i}^{w}}\frac{1}{2}{\left(\tau_{i}^{w}\right)}^{2};\\ \mathrm{subject}\;\mathrm{to}\text{:}\;:\\ \;\tau_{i}^{w}\geq 0;\\ \;|\tau_{zi}-\tau_{i}^{w}-\tau_{i}^{in}|\geq \tau_{safety}. \end{array}\right.$$

Note that (24) is the same as Problem (4) in which $x_{1}=\tau_{i}^{w}$, $c=\tau_{zi}-\tau_{i}^{in}$, $x_{s}=\tau_{safety}$. The solution of this problem is determined according to (16) and (17), which yields $\tau_{i}^{w*}=0$ if $\tau_{safety}\leq \tau_{zi}-\tau_{i}^{in}$ or $\tau_{safety}\leq -\left(\tau_{zi}-\tau_{i}^{in}\right)$. In the first case, the vehicle $i\in Veh_{IN}$, being the vehicle that wants to enter the roundabout through entry $E_{i}$, will enter in front of vehicle $v_{zi}\in Veh_{IN_{zi}}$. In the second case, the vehicle $v_{zi}$ will pass first in front of entry $E_{i}$ and the vehicle *i* will enter the roundabout behind it. In the third case, if $\tau_{safety}\geq -\left(\tau_{zi}-\tau_{i}^{in}\right)$, the solution will be $\tau_{i}^{w*}=\tau_{safety}+\left(\tau_{zi}-\tau_{i}^{in}\right)$. In this last case, the vehicle will wait for the vehicle that is already in the intersection to pass first; after that, it will enter the roundabout. The distance between vehicles when vehicle *i* enters the roundabout is $\delta_{safety}=v/\tau_{safety}$.

**Case 3**: Next, the case in which there are $n_{z}$ vehicles inside the roundabout that will pass in front of entry $E_{i}$ through which vehicle *i* wants to enter is solved. Considering $\mathrm{Card}(Veh_{IN})=1$, $\mathrm{Card}(Veh_{IN_{ji}})=0$, $\mathrm{Card}\left(Veh_{IN_{zi}}\right)=n_{z}$, Problem 6 becomes:

(25) $$\left\{\begin{array}{c} \min_{\tau_{i}^{w}}\left(J\left(\tau_{i}^{w}\right)\right)=\min_{\tau_{i}^{w}}\frac{1}{2}{\left(\tau_{i}^{w}\right)}^{2};\\ \mathrm{subject}\;\mathrm{to}\text{:}\;\\ \;\tau_{i}^{w}\geq 0;\\ \;|\tau_{z\overset{ˇ}{z}i}-\tau_{i}^{w}-\tau_{i}^{in}|\geq \tau_{safety},\;\overset{ˇ}{z}=\bar{1,n_{z}}. \end{array}\right.$$

In this case, it is assumed that vehicle $v_{z\overset{ˇ}{z}i}$ is in front of vehicle $v_{z(\overset{ˇ}{z}+1)i}$, $\overset{ˇ}{z}=\bar{1,n_{z}-1}$, and then the problem can be solved using Algorithm  1.
**Algorithm 1:** Case 3
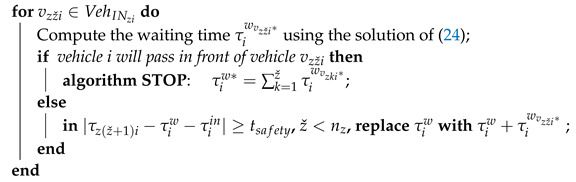


Analysing the proposed solution given by Algorithm 1 it can be noticed that for vehicle *i* from entry $E_{i}$, the CM will compute the waiting time required to ensure a safe entry, by initially considering the first vehicle already in the roundabout, which will pass in front of $E_{i}$. If vehicle *i* can enter the intersection in front of this vehicle, the algorithm will stop. Otherwise, it will compute the waiting time considering the next vehicle and the waiting time obtained for the first vehicle and so on.

**Case 4**: The last case, which is the most general and realistic one, represents the situation in which $n_{k}\leq n$ vehicles want to enter the intersection, each vehicle will enter through a unique entry and in front of these entries will pass $n_{zi}$ vehicles that are already in the roundabout, also $n_{ji}$ vehicles from those $n_{k}$ vehicles will pass if front of entry $E_{i}$, $i=\bar{1,n_{k}}$. Considering that $\mathrm{Card}\left(Veh_{IN}\right)=n_{k}$, $\mathrm{Card}\left(Veh_{IN_{ji}}\right)=n_{ji}$, $\mathrm{Card}\left(Veh_{IN_{zi}}\right)=n_{zi}$, Problem 6 becomes:

(26) $$\left\{\begin{array}{c} \min_{\left[\tau_{1}^{w},\ldots ,\tau_{n_{k}}^{w}\right]}\left(J\left(\left[\tau_{1}^{w},\ldots ,\tau_{n_{k}}^{w}\right]\right)\right)=\min_{\left[\tau_{1}^{w},\ldots ,\tau_{n_{k}}^{w}\right]}\left(\frac{1}{2}\sum_{i=1}^{n_{k}}{\left(\tau_{i}^{w}\right)}^{2}\right);\\ \mathrm{subject}\;\mathrm{to}\text{:}\;:\\ \;\;t_{i}^{w}\geq 0;\\ \;\;|\tau_{j_{i}}^{in}+\tau_{j_{i}}^{w}+\tau_{j_{i}i}-\tau_{i}^{in}-\tau_{i}^{w}|\geq \tau_{safety};\\ \;\;|\tau_{z\overset{ˇ}{z}i}-\tau_{i}^{w}-\tau_{i}^{in}|\geq \tau_{safety};\\ \;\;\overset{ˇ}{z}=\bar{1,n_{zi}},i=\bar{1,n_{k}},j_{i}=\bar{1,n_{ji}}. \end{array}\right.$$

Furthermore, in this case, it is assumed that vehicle $v_{z\overset{ˇ}{z}i}$ is in front of vehicle $v_{z(\overset{ˇ}{z}+1)i}$, $\overset{ˇ}{z}=1,\cdots ,n_{zi}-1$, $i\in \{1,2,\cdots ,n_{k}\}$, then the problem can be solved using Algorithm 2.
**Algorithm 2:** Case 4
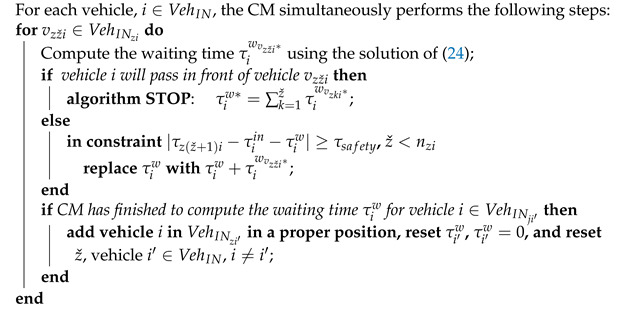


When CM finishes computing the waiting time $\tau_{i}^{w}$ for vehicle $i\in Veh_{IN_{ji^{\prime }}}$, then vehicle *i* will be added in $Veh_{IN_{zi^{\prime }}}$ in a proper position, i.e., the vehicles from array $Veh_{IN_{zi^{\prime }}}$ are in the same order as they will pass in front entry $E_{i}^{\prime }$. In the case in which $\mathrm{Card}(Veh_{IN_{zi}})=0$, $i\in Veh_{IN}$, then the waiting time $\tau_{i}^{w*}=0$ because there are no vehicles inside the roundabout to pass in front of entry $E_{i}$, i.e., the case illustrated by Problem 2.

## 5. Results

In this section, the results obtained with the proposed solution are presented. The simulated intersection has four entries and four exits with the parameters illustrated in Table 1.

The parameters of the roundabout directly influence the performances of the solution as follows: (i) the maximum number of vehicles in the roundabout depends on its radius and the distances between the vehicles, (ii) the velocities of the vehicles determine the time in which they cross the intersection and, (iii) the safety time, which influences the safety distance, determines the minimum distance between vehicles.

The waiting times are computed using the solution presented in Section 4. To simulate the movements of vehicles through the intersection, they were modelled using a point model that describes the longitudinal and the lateral dynamics of the vehicles. The interested reader is referred to a more detailed description in [32]. Note that the CM, knowing about the velocity of the vehicles that are entering the roundabout, can always estimate their position through the roundabout.

**Figure 3:**
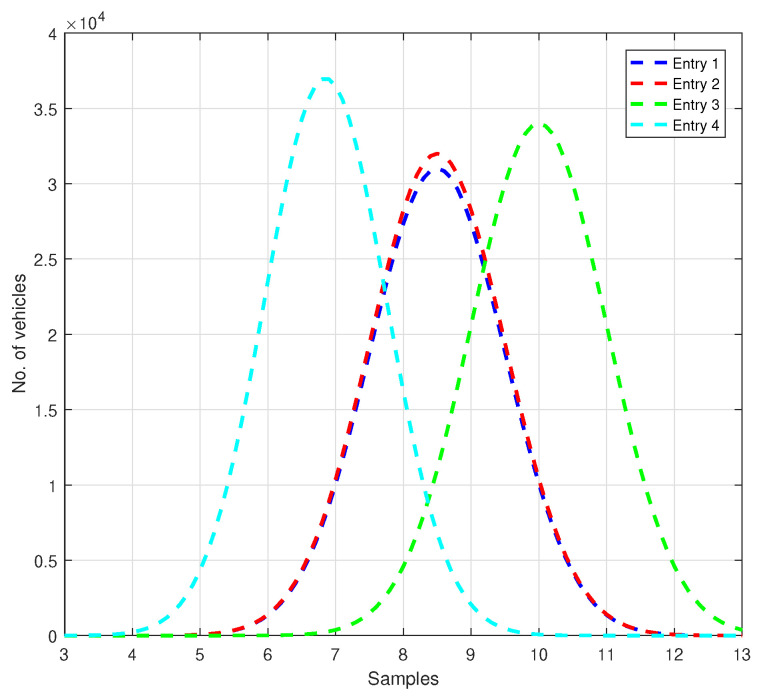
The rates of generation for the vehicles analysed during an entire day.

The rates used to generate the vehicles that want to cross the roundabout during an entire day have a Gaussian distribution and are illustrated in Figure 3. For the first entry, the average rate of the vehicles that want to enter is 1vehicle/4.25 s, for the second—1vehicle/4.25 s, for the third—1vehicle/5 s, and for the fourth entry—1vehicle/3.42 s. From this analysis, it results that through entry 2, fewer vehicles want to pass when compared to entry 4 and, at the same time, more vehicles than through entry 3.

**Figure 4:**
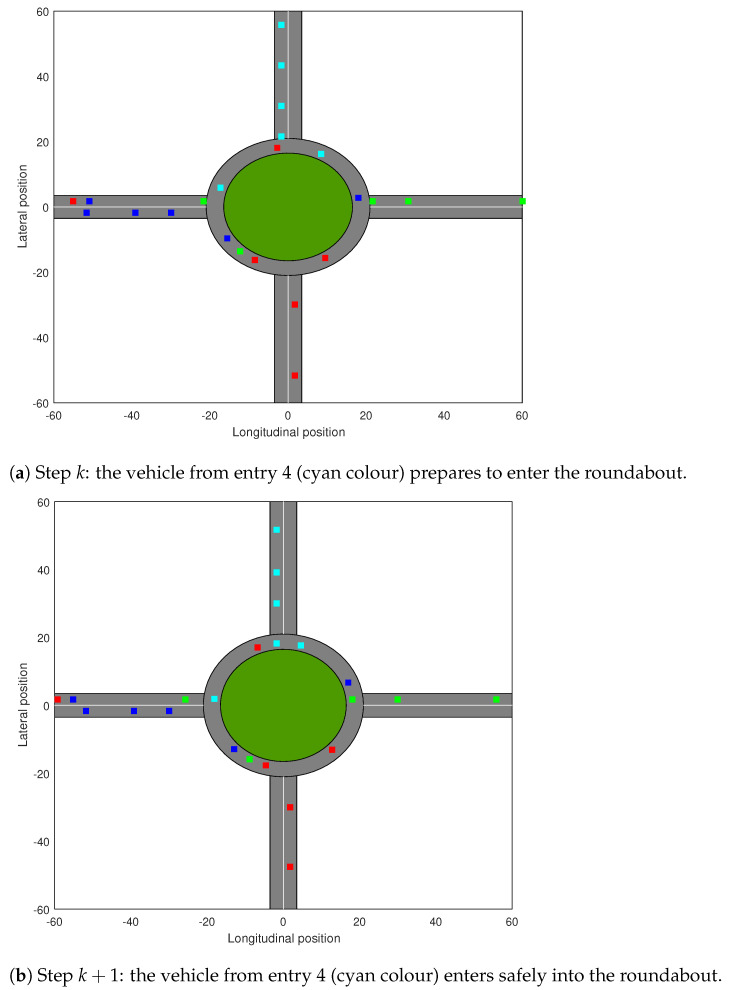
Entry 4—simulation detail snapshots.

Figure 4 illustrates two consecutive steps (upper and lower sub-figures), from which one can notice that in front of entry 4 (illustrated with cyan colour), a vehicle from entry 2 (represented with red colour) passes. It can be observed that the CM succeeds to compute a waiting time for the vehicle of entry 4 to avoid the collision and to enter the roundabout at a safe distance.

To fully test the solution, a simulation of 1 h was performed. The results are illustrated in Table 2 and represent the minimum, maximum, and the average waiting times and the number of vehicles that entered the roundabout. Furthermore, for comparison, Table 3 illustrates the results obtained with the solution from our previous work [32]. In that paper, the same problem, (6), was solved but using the Matlab function fmincon. It can be noticed that the analytical solution proposed in the current work allows more vehicles to cross the intersection compared to the solution based on the fmincon function, while the average waiting time is four times smaller for the analytical solution.

**Figure 5:**
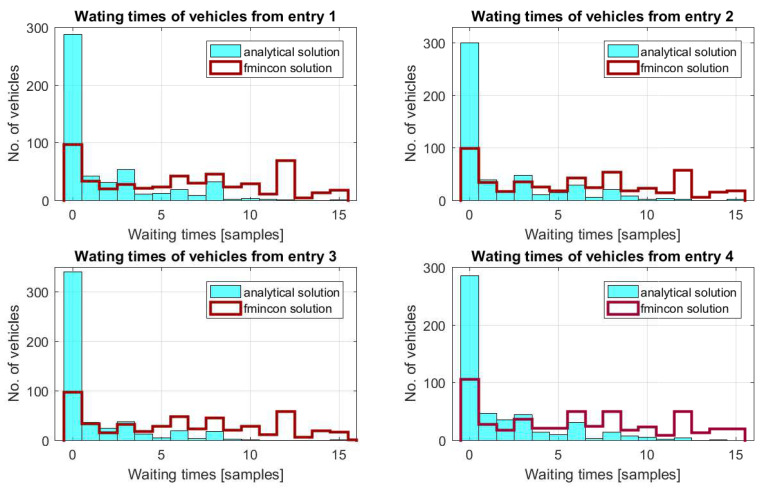
Waiting times of the vehicles.

**Figure 6:**
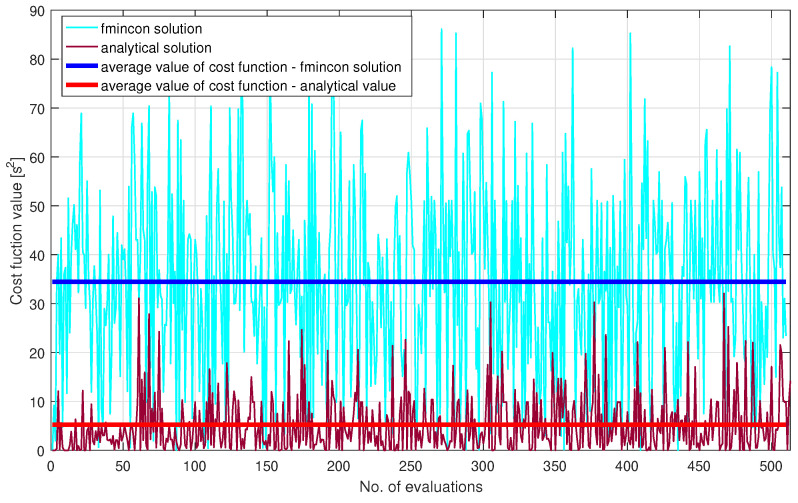
Values of the cost functions.

In Figure 5, the waiting times of the vehicles that cross the roundabout are illustrated. Analysing these figures, it can be observed that the values of the waiting times obtained with the analytical solution are lower compared to those obtained by the fmincon solution provided by Matlab. From these figures and Table 2 and Table 3, it can also be observed that the number of vehicles that cross the roundabout has increased. Moreover, in Figure 6, one can observe that a better result is obtained for the solution presented in Section 4.

**Figure 7:**
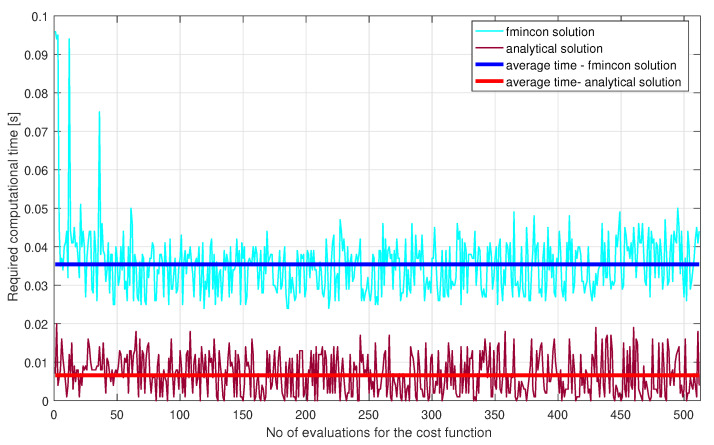
Required computational time.

It can be noticed that the results obtained using the multivariable optimisation-based analytical solution proposed in this paper outperforms the results obtained in our previous work [32]. Since the average waiting times for each entry has decreased, this leads to an increased number of vehicles that crossed the roundabout. The simulation was done using Matlab R2016a, on a system equipped with a Core i7 processor and 8GB memory RAM. The computational times for both the proposed solution and the comparative fmincon solution from [32] are illustrated in Figure 7. Because the required computational average time needed by the CM, implemented based on the proposed analytical solution, to obtain the waiting time is smaller than the average time needed by the fmincon function, i.e., 0.0066 s < 0.035 s, results that the proposed analytical solution can be implemented on a real-time equipment with minimum costs. Another advantage is given by its overall implementation simplicity, when compared to the fmincon function.

## 6. Conclusions

The main contribution of this paper is represented by the proposed cyber-physical framework based on an analytical solution for a roundabout intersection with *n* entries and exits with the aim of minimising the waiting times of the vehicles entering the intersection and ensuring a safe crossing. The results show that the proposed methodology is robust and can be easily implemented on an embedded device for real-time operation. The robustness of the solution is given by the capability of the CM to compute the optimal waiting times, independent of: (i) the number of entries/exits of the roundabout, (ii) the imposed velocity for the vehicles or (iii) the number of vehicles that want to cross the intersection.

Future work will focus on improving the proposed solution to consider roundabouts with multiple lanes, vehicles moving with variable velocities and mixed traffic.

## Figures and Tables

**Table 1:** Roundabout parameters.

Parameter	Description	Value
*R*	Roundabout radius	20 m
*d*	Control zone length	10 m
$d_{2}$	Distance between waiting-point and the roundabout	10 m
*l*	Lane width	3.5 m
*v*	Imposed vehicle’s velocity	8.33 m/s
$T_{s}$	Sampling time	0.5 s
$\tau_{safety}$	Safety time	1 s

**Table 2:** Waiting times—analytical solution.

Entry No.	Min. $t_{\mathrm{wait}}$	Max. $t_{\mathrm{wait}}$	Average $t_{\mathrm{wait}}$	No. of Entered Vehicles
Entry 1	0	15$T_{s}$	1.797$T_{s}$	769
Entry 2	0	15$T_{s}$	1.795$T_{s}$	784
Entry 3	0	15$T_{s}$	1.26$T_{s}$	663
Entry 4	0	15$T_{s}$	1.77$T_{s}$	920

**Table 3:** Waiting times—fmincon solution.

Entry No.	Min. $t_{\mathrm{wait}}$	Max. $t_{\mathrm{wait}}$	Average $t_{\mathrm{wait}}$	No. of Entered Vehicles
Entry 1	0	15$T_{s}$	6.08$T_{s}$	513
Entry 2	0	15$T_{s}$	6.42$T_{s}$	513
Entry 3	0	16$T_{s}$	6.16$T_{s}$	513
Entry 4	0	16$T_{s}$	6.28$T_{s}$	513

## Data Availability

Not applicable.
